# Predicting lncRNA-miRNA Interaction *via* Graph Convolution Auto-Encoder

**DOI:** 10.3389/fgene.2019.00758

**Published:** 2019-08-29

**Authors:** Yu-An Huang, Zhi-An Huang, Zhu-Hong You, Zexuan Zhu, Wen-Zhun Huang, Jian-Xin Guo, Chang-Qing Yu

**Affiliations:** ^1^College of Electronics and Information Engineering, Xijing University, Xi’an, China; ^2^Department of Computer Science, City University of Hong Kong, Kowloon, Hong Kong; ^3^College of Computer Science and Software Engineering, Shenzhen University, Shenzhen, China

**Keywords:** LncRNA–miRNA interactions, graph convolution network, computational prediction model, regulation network, system biology model

## Abstract

The interaction of miRNA and lncRNA is known to be important for gene regulations. However, the number of known lncRNA-miRNA interactions is still very limited and there are limited computational tools available for predicting new ones. Considering that lncRNAs and miRNAs share internal patterns in the partnership between each other, the underlying lncRNA-miRNA interactions could be predicted by utilizing the known ones, which could be considered as a semi-supervised learning problem. It is shown that the attributes of lncRNA and miRNA have a close relationship with the interaction between each other. Effective use of side information could be helpful for improving the performance especially when the training samples are limited. In view of this, we proposed an end-to-end prediction model called GCLMI (Graph Convolution for novel lncRNA-miRNA Interactions) by combining the techniques of graph convolution and auto-encoder. Without any preprocessing process on the feature information, our method can incorporate raw data of node attributes with the topology of the interaction network. Based on a real dataset collected from a public database, the results of experiments conducted on k-fold cross validations illustrate the robustness and effectiveness of the prediction performance of the proposed prediction model. We prove the graph convolution layer as designed in the proposed model able to effectively integrate the input data by filtering the graph with node features. The proposed model is anticipated to yield highly potential lncRNA-miRNA interactions in the scenario that different types of numerical features describing lncRNA or miRNA are provided by users, serving as a useful computational tool.

## Introduction

In recent years, the knowledge of the role of RNA in gene regulation has emerged from the advances in next-generation sequencing technologies, allowing a deeper and more comprehensive study on full transcriptomes of organisms. It is demonstrated by the ENCODE project that in mammals noncoding RNA could constitute a substantial majority of transcripts within the genome (Science, 2004). There is as much as 98% of the whole human genome encoding for noncoding transcripts, most of which are processed to generate small noncoding RNA such as miRNA, or long noncoding RNA (lncRNA).

Even though the current understanding of lncRNA functions is still limited, it is revealed that they are key regulators of multiple biological processes through a complex mechanism in which their modular structure permits them to interact with specific proteins, RNA, and DNA (Wapinski and Chang, 2011). On the other hand, miRNAs post-transcriptionally regulate the expression of their target genes. Accumulating studies are showing that, similar to the protein-coding genes, both of these two types of noncoding RNA influence almost all aspects of biology (Lu et al., 2005). The aberrant expression level of noncoding RNAs appears to be one of the initiating factors of different types of disease including cancers (Lewis et al., 2003; Calin and Croce, 2006).

A number of studies have begun to uncover the interactions between miRNA and lncRNA and more and more details about the influence of miRNA on lncRNA function is now coming into view (Tay et al., 2014). In some cases, miRNA triggers lncRNA decay. In other cases, lncRNA acts as miRNA sponges/decoys, or competes with miRNA for binding mRNAs or generate miRNAs. Recently, the hypothesis of competing endogenous RNA (ceRNA) has been proposed and become a mainstream view for explaining the interaction between lncRNA and miRNA (Salmena et al., 2011). Specifically, lncRNA competes with pseudogenes, circular RNAs and messenger RNAs for binding or sequestering microRNAs from the same pool through matching the miRNA response elements (MREs). Considering that both lncRNA and miRNA are keys to regulate gene expression and they interact with each other, it is not unexpected that their relationship in interaction network is firmly regulated. Understanding the lncRNA-miRNA interactions networks governing the initiation and development of diverse diseases is essential but remains largely uncompleted (Karreth and Pandolfi, 2013).

LncRNAs and miRNAs interact with each other forming a huge and complex regulation network for controlling gene expression on transcriptional, post-transcriptional, and post-translational levels. Through this multi-level regulation, these two vast families of noncoding RNAs are involved in almost all aspects of cell cycles including cell division, senescence, differentiation, stress response, immune activation, and apoptosis (Shi et al., 2013). In view of this, interactions of noncoding RNAs on the regulation network have attracted widespread attention in medical research (Huang et al., 2016a). A comprehensive understanding of the molecular and cellular effects of such noncoding interaction can offer great insight into the disease mechanism at a molecular level. Noncoding RNAs in those interactions newly discovered to be associated with a specific disease can be regarded as potential diagnostic markers and therefore is of high value in therapeutic approaches.

Some efforts have been made to design a computational method to meet the emerging need for an accurate prediction of lncRNA-miRNA interactions on a large scale. One popular direction is to do statistical analysis on the data collected from biological experiments. For example, Sumazin et al. attempted to construct a miRNA-mediated network of coding and non-coding RNA interactions for inferring the key dysregulation of ncRNA expression in pathogenesis (Sumazin et al., 2011). The algorithm of Hermes they proposed for such network calculates the statistical significance of each RNA-miRNA-RNA triplet by matching the expression profiles of gene and miRNAs in glioblastoma. Similarly, Paci et al. and Conte et al. construct lncRNA-miRNA-RNA interaction network by calculating so-called sensitivity correlation which denotes the difference between Pearson correlation coefficient and partial correlation coefficient for each triplet obtained from the breast cancer data (Paci et al., 2014; Conte et al., 2017). To investigate the underlying roles of lncRNA in the diseases of prostate cancer and lung adenocarcinoma, Du et al. and Sui et al. integrate different types of attribute data of RNA to construct a regulatory network in which lncRNAs centrally mediate miRNAs (Du et al., 2016; Sui et al., 2016). All of these methods are designed based on statistics measure and their statistics analysis is for a specific type of disease. To identify the noncoding RNA-mediated sponge regulatory network in various diseases recorded in TCGA and UCEC, Wang et al. construct lncRNA-miRNA-gene triplet networks yielded by prediction algorithms. Based on such constructed networks, hypothesis testing approach is implemented for predicting those triplets associated with diseases (Wang et al., 2015).

Another direction for predicting lncRNA-miRNA is based on matching seed sequences. Most computational tools of such type, such like TargetScan, miRanda and RNAhybrid, aim at predicting miRNA targets selecting evolutionarily conserved microRNA binding sites (Zheng et al., 2017). However, it is pointed out by Natalia et al. that prediction using these methods could be of high false positives and often biologically irrelevant (Pinzón et al., 2017). They show that the interaction between lncRNAs and miRNAs is dose-sensitivity. In view of this, it is hardly to predict miRNA target only using the sequence information as they are not always dose-sensitive enough to be functionally regulated by miRNAs.

The past decade has witnessed the exponential growth of noncoding RNA expression profiling data in cancers but the number lncRNA-miRNA interactions underlying such big data is still limited (Zheng et al., 2016b). Considering that different attributes of noncoding RNAs are being continuously updated, the big data about noncoding RNAs poses significant challenge for data analysis and integration, which is important for predicting new links on the current sparse lncRNA-miRNA interaction network. In taking forward this area of work, some methods based on machine learning have been proposed. Huang et al. propose the first prediction model for inferring lncRNA-miRNA on a large scale. Specifically, the EPLMI model uses a network diffusion method on weighted networks associated with expression profiles, sequence information and biological function (Huang et al., 2018). The basic assumption of this method is based on the finding that miRNAs of similar patterns tend to interact with similar lncRNA and vice versa. However, how to define the similarity among noncoding RNAs based on their expression profile is still an open problem. EPLMI model use Person correlation coefficients to compute such similarity, which means it assumes each element in the noncoding RNA features equally contributes to the similarity score. However, it would be inappropriate for the nature of its mechanism.

In recent years, the advance of deep learning fuels the widespread use of data mining in many different science areas including bioinformatics (Li et al., 2016). Specially, graph convolution comes to be a powerful and popular technique in data mining for graph-based data. It proves to be powerful for its ability to automatically learn latent features from an end-to-end model structure. The hidden layers within the model thus are able to extract meaningful information from the raw input data. In this work, we introduce the technique of graph convolution into the model of autoencoder for building an end-to-end deep learning prediction model called GCLMI for inferring new lncRNA-miRNA interaction on a large scale. Specifically, two different layers are respectively designed to encode and decode the raw feature of each nodes on the input graph. As a result, the decoder can yield a fully-connected network in which the predicted score of each link represent the confidence coefficient of it to be true. Different from the sequence-based algorithms which only consider the sequence information, GCLMI is a network-based algorithm which considers the known lncRNA-miRNA interactions along with the expression levels of lncRNA and miRNA. In addition, GCLMI aims to compute the possibility of a lncRNA-miRNA pair to be interactive in biological processes while sequence-based tools aim to predict the binding sites of miRNA in transcripts.

To evaluate the prediction performance of the proposed model, we implement it in a real dataset of lncRNA-miRNA interactions. By using the frameworks of 2-fold, 5-fold and 10-fold cross validation, the prediction model yielded average AUCs of 0.8492+/−0.0013, 0.8567+/−0.0009 and 0.8590+/−0.0005, respectively. The results of a series of comparison experiments show that the model we present is superior to some methods previously proposed. In addition, the results also illustrate the ability of graph convolution to integrate the raw features of nodes and the topology of graph. The experimental results overall prove that the deep learning-based model we proposed is reliable to yield accurate results and robust to parameter settings. It is anticipated that the proposed model could be served as a useful computational tool for predicting large-scale lncRNA-miRNA interactions in the scenario that know lncRNA-miRNA interactions along with their expression profile are given by users.

## Method

### Materials

The number of known lncRNA-miRNA interactions is still limited and expression profiles of lncRNA and miRNA are often be used for inferring those lncRNA-miRNA pairs of high correlations. Although the number such results is huge, but they are not truly confirmed by the experiments based on CLIP-Seq techniques and therefore would negatively affect the prediction results (Zheng et al., 2016a). To obtain the ground true data resource for our prediction, we collected a dataset of lncRNA-miRNA interactions that are experimentally confirmed from the lncRNASNP database (version v1.0). lncRNASNP is a comprehensive database for lncRNA and provides different kinds of relevant data resource including lncRNA expression profiling, expanded lncRNA-associated diseases, and noncoding variants in lncRNAs (available at http://bioinfo.life.hust.edu.cn/lncRNASNP). The database matches the IDs of lncRNAs and integrates data from different public databases including that of lncRNA-miRNA interactions from starBase. Eight thousand ninety-one pairwise interactions including 780 types of lncRNA and 275 types of miRNA are totally recorded (Gong et al., 2014). Such interactions have already been verified *via* laboratory examination and therefore are of high confidence.

There are different types of data able to be used as the features of lncRNA and miRNA, such as sequence information of nucleotides, expression profiles, target genes and predicted functional annotations (Zheng et al., 2018). Sequence information is complete for both of lncRNA and miRNA but is too complicated for model to learn as it is nominal and of different length for different types. The links between noncoding RNA and target genes would be meaningful for inferring their interactions but such information is scarce and incomplete for many types of them. The functional annotation of noncoding RNA is important for understanding the characters of one noncoding RNA and many works have been made to inferring them by considering different types of complementary data. However, such information is yielded by prediction algorithms with additional assumptions and therefore is possible to cause computation bias on further prediction models. The superiority of expression profile data to others has been illustrated in our previous work by the experimental results (Huang et al., 2018). For such reason, we only focus on the expression profiles of noncoding RNAs in this work.

To collect the expression profile data of lncRNAs, we match the ids of lncRNAs from two different databases of lncRNASNP and NONCODE (http://www.noncode.org/) (Bu et al., 2011). For 780 types of lncRNA recorded in the lncRNASNP database, 450 of them are successfully matched with their expression profiles. The data of expression profile for each type of lncRNA present the expression level of it in 22 different human tissues or cell lines. For the features of miRNAs, we collect them from the microRNA.org database (http://www.microrna.org/) (Betel et al., 2008). As a result, the ids of 230 out of 275 types of miRNA are successfully converted from lncRNASNP into microRNA.org database. Each entry of miRNA expression profiles consists of 172 values describing the expression level of such miRNA in 172 various tissues and cell lines in human body.

### Graph Convolution

**Figure 1:**
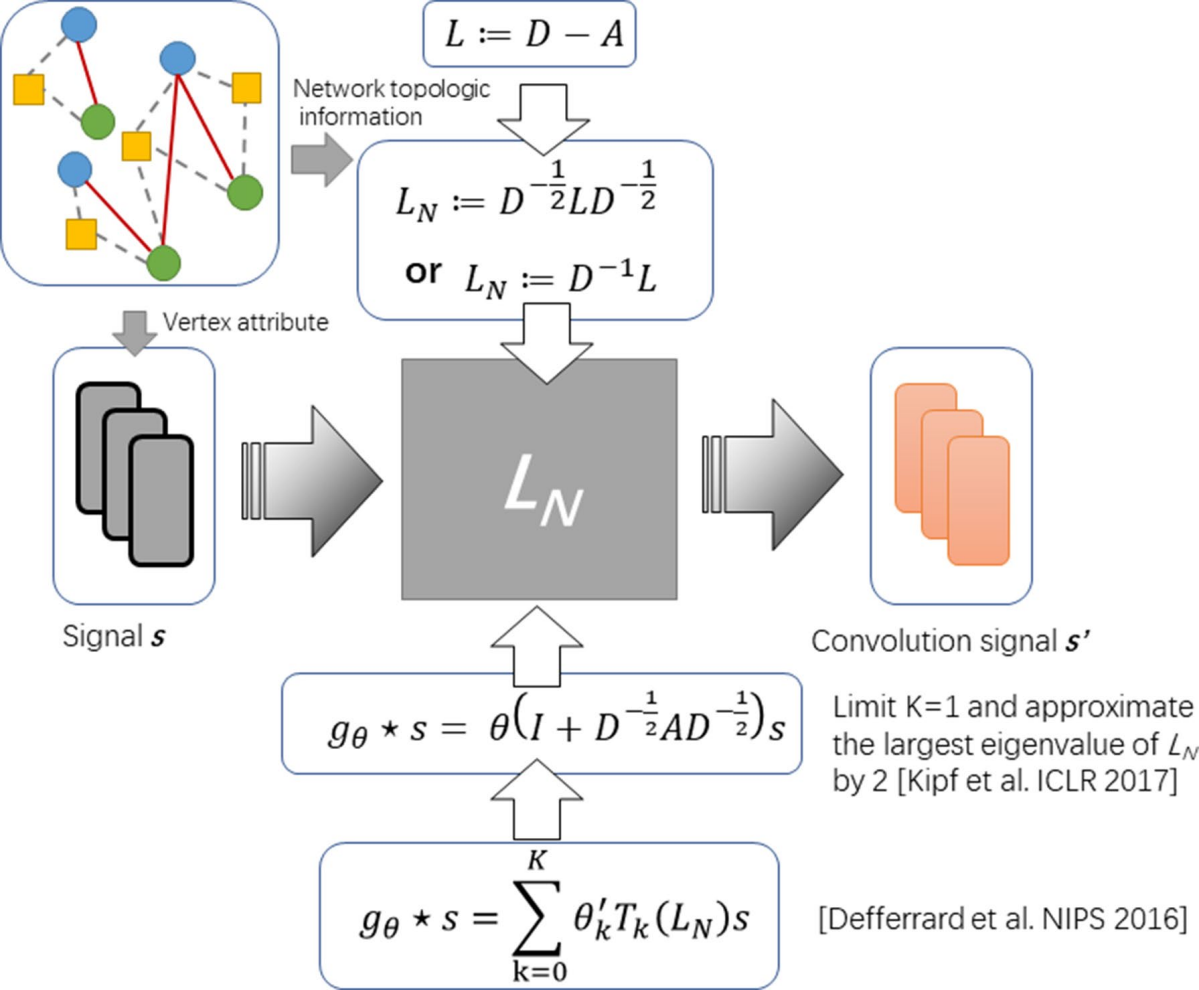
The diagram of spectral graph convolution.

It is still an open problem to define the convolution operator on a graph and generalizing convolutional neural networks (CNNs) to arbitrary graphs comes to be a recent area of interest (Kearnes et al., 2016). So far, the approaches with graph convolution could be categorized into two types: i) one is based on definitions of spatial convolution and ii) the other is based on the graph spectral theory. The latter is more popular and is elegantly defined as a multiplication in the graph Fourier domain. The spectral framework was first to be introduce in the context of graph CNNs by Bruna et al. (Bruna et al., 2013). Along this direction, Kipf et al. propose an optimization strategy based on approximal first-order on the spectral filters, reducing its complexity from O(n^2^) to O(|ε|) (see **Figure 1**) (Kipf and Welling, 2016).

To formulate the operator of spectral convolution on graph, given an adjacent matrix A of graph *G* with its Laplacian *L* := *D* – *A* and attributes of each node on graph (say s), Defferrard et al. (2016) propose spectral graph convolution to filter s by a non-parametric kernel *g*_θ_(Λ) = *diag*(θ), where θ is a vector of Fourier coefficients. Given *L* can be decomposed by L = UΛUT, where Λ is the diagonal matrix of eigenvalues and U is eigenvector matrix, such operator could be defined as

(1) $$g_{\theta }*s=Ug_{\theta }U^{T}s$$

Approximating the spectral filter by using a truncated expansion in terms of Chebyshev polynomials T_k_(s) up to K^th^ order, the definition is as follows:

(2) $$g_{\theta }*s\approx \sum_{k=0}^{K}\theta_{k}^{\prime }´T_{k}(L_{N})s$$

where *T* *_k_* denotes Chebyshev polynomials and *θ*ʹ is a vector of Chebyshev coefficients. Considering the complexity of computing L is as large as O(n^2^), Kipf and Welling. (2016) further simplified this definition by limiting K = 1 and approximating the largest eigenvalue of L by 2. The convolution operator comes to be:

(3) $$g_{\theta }*s=\theta (I+D^{-\frac{1}{2}}AD^{-\frac{1}{2}})s$$

By introducing the renormalization tricks: $I_{n}+D^{-\frac{1}{2}}AD^{-\frac{1}{2}}\to {\tilde{D}}^{-\frac{1}{2}}\tilde{A}{\tilde{D}}^{-\frac{1}{2}}$ with Ã = *A + I* *_N_* and ${\tilde{D}}_{ii}=\sum_{j}{\tilde{A}}_{ij}$, formula (3) can be simplified as:

(4) $$g_{\theta }*s=\theta {\tilde{D}}^{-\frac{1}{2}}\tilde{A}{\tilde{D}}^{-\frac{1}{2}}s$$

In this work, we follow this definition as formula 4 for design our deep learning model based on the graph convolution.

### GCLMI: An Auto-Encoder Prediction Model for lncRNA-miRNA Interactions

In this work, we cast the prediction task for lncRNA-miRNA interactions as a link prediction problem on a heterogeneous bipartite graph. Consider an adjacent matrix of such graph *M* of shape *N* *_l_* × *N* *_m_*, where *N* *_l_* is the number of lncRNA nodes and *Nm* is the number of miRNA nodes. Entry *M* *_ij_* in this matrix encode either the interaction between *i*-th type of lncRNA and *j*-th type of miRNA is identified by biological experiments or not. The task of prediction can be considered as referring the value of unobserved entries in M using semi-supervised learning on the observed ones.

**Figure 2:**
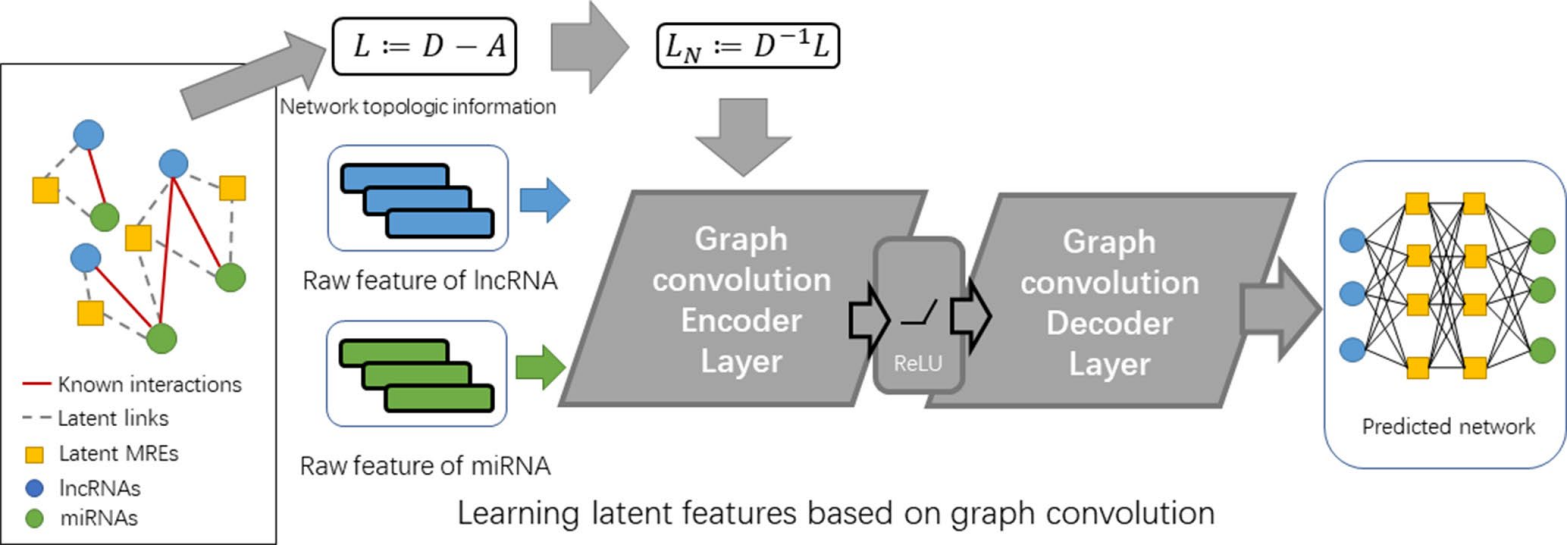
The flowchart of GCLMI.

In an equivalent picture, we can also represent the interaction data by an undirected graph *G* = (ν, ε, *X* *_l_*, *X* *_m_*), where *X* *_l_* and *X* *_m_* are the feature matrices for the lncRNA nodes and miRNA nodes, respectively. The goal is to learn embedding features for lncRNAs and miRNAs *E* by building a graph-based encoder [*E* *_l_*, *E* *_m_*] = *f* *_en_* (ν, ε, *X* *_l_*, *X* *_m_*) and predicting new links by building a decoder *M*’ = *f*de (*E* *_l_*, *E* *_m_*). *E* *_l_* and *E* *_m_* are the feature matrices for lncRNAs and miRNA with shapes of *N* *_l_* × *L* and *N* *_m_* × *L*, respectively (see **Figure 2**).

To this aim, our proposed model is composed by two layers of different types: i) an encoder layer for filtering node features of lncRNA and miRNA on the graph of their interaction network and ii) a decoder layer for predicting fully-collected interaction network using the embedding features learned from the former layers.

The inputs of encoder layer include the feature matrixes of lncRNA and miRNA (i.e. *F* *_l_* and *F* *_m_*) and the adjacent matrix of known lncRNA-miRNA interaction network (i.e. M). In order to integrate the features of lncRNA and miRNA into one input matrix, an expanded matrix *X* is constructed based on *F* *_l_* and *F* *_m_* as follows:

(5) $$X=\left[\begin{array}{cc} F_{l} & 0\\ 0 & F_{m} \end{array}\right]$$

Accordingly, the adjacent matrix of known lncRNA-miRNA interaction network is expanded as:

(6) $$A=\left[\begin{array}{cc} 0 & M\\ M^{T} & 0 \end{array}\right]$$

Based on the above two input matrixes, we compute a graph convolution matrix G according to formula 4:

(7) $$G=X_{rw}\left(I+D^{-\frac{1}{2}}AD^{-\frac{1}{2}}\right)$$

The hidden layer is then built based on G by introducing its weight matrix We and bias matrix Be. With ReLU as the activation function, the output E of the encoder layer would be as follows:

(8) $$E=\mathrm{ReL}\text{U}\left(G\cdot W_{e}+B_{e}\right)=\left[\begin{array}{c} E_{l}\\ E_{m} \end{array}\right]$$

where the trainable weight matrix *W* *_e_*∈(*D* *_l_* + *D* *_m_*) × *N* *_e_* transforms the convolution matrix G into a hidden matrix *E*. *N* *_e_* denotes the number of latent factors and is set manually. The output layer learned from the encoder layer is a projection from the space of raw features into a hidden space with lower rank. As lncRNA and miRNA are known to interact with each through the MRE on transcripts, the design of hidden accords with the nature that lncRNA, MRE, miRNA is associated in a three-layer relation network.

The output of the encoder layer has two components, which are the matrix of the embedding feature matrix of lncRNA *E* *_l_* and that of miRNA *E* *_m_*. Introducing a trainable weight matrix Wd, the decoder layer is then built based on these separate matrixes of the same raw dimension as follows:

(9) $$M^{\prime }=E_{l}W_{d}E_{m}^{T}$$

The output matrix *M*’ clearly has the same shape out the input matrix *M*. As matrix *M*’ is numerical, it describes the weight of links in a fully-connected network. All lncRNA-miRNA pairs with value of 0 in matrix M would be assigned a predicted value by the decoder. Those pairs with high predicted scores are anticipated to more possibly be connected.

To train the model of GCLMI in a semi-supervised learning manner, we use the strategy of negative sampling. Specifically, in each epoch of training process, we randomly select a fixed number of negative samples from the unlabeled lncRNA-miRNA pairs. The loss function of our training is defined as follows:

(10) $$\begin{array}{l} ℒ=\sqrt{\frac{\sum ij;\Omega_{p,ij}=1or\;\Omega_{n,ij}=1{\left(M_{ij}^{\prime }-M_{ij}\right)}^{2}}{\sum ij\left(\Omega_{p,ij}+\Omega_{n,ij}\right)}}+\frac{1}{2}||W_{e}|{|}^{2}\\ \;+\frac{1}{2}||W_{d}|{|}^{2}+\frac{1}{2}||B_{e}|{|}^{2} \end{array}$$

where the matrices $\Omega_{p}\in {\left\{0,1\right\}}^{N_{l}\times N_{m}}$ and $\Omega_{n}\in {\left\{0,1\right\}}^{N_{l}\times N_{m}}$ denote the masks for positive samples and the negative samples from random sampling, respectively. The first term in equation (10) aims to minimize the prediction error and the second and the third term define the constraint on the weight matrix in encoder and decoder, respectively. As negative sampling is implemented for training, in each epoch the Ω_n_ would be randomly generated in which the number of “1” would be fixed as a specific percentage of the number of positive samples. Hence, we would only optimize over the positive samples if we set this percentage as 0 or optimize over the positive samples and partial negative samples otherwise.

## Results and Discussion

### Evaluation of Graph Convolution’s Effectiveness

**Figure 3:**
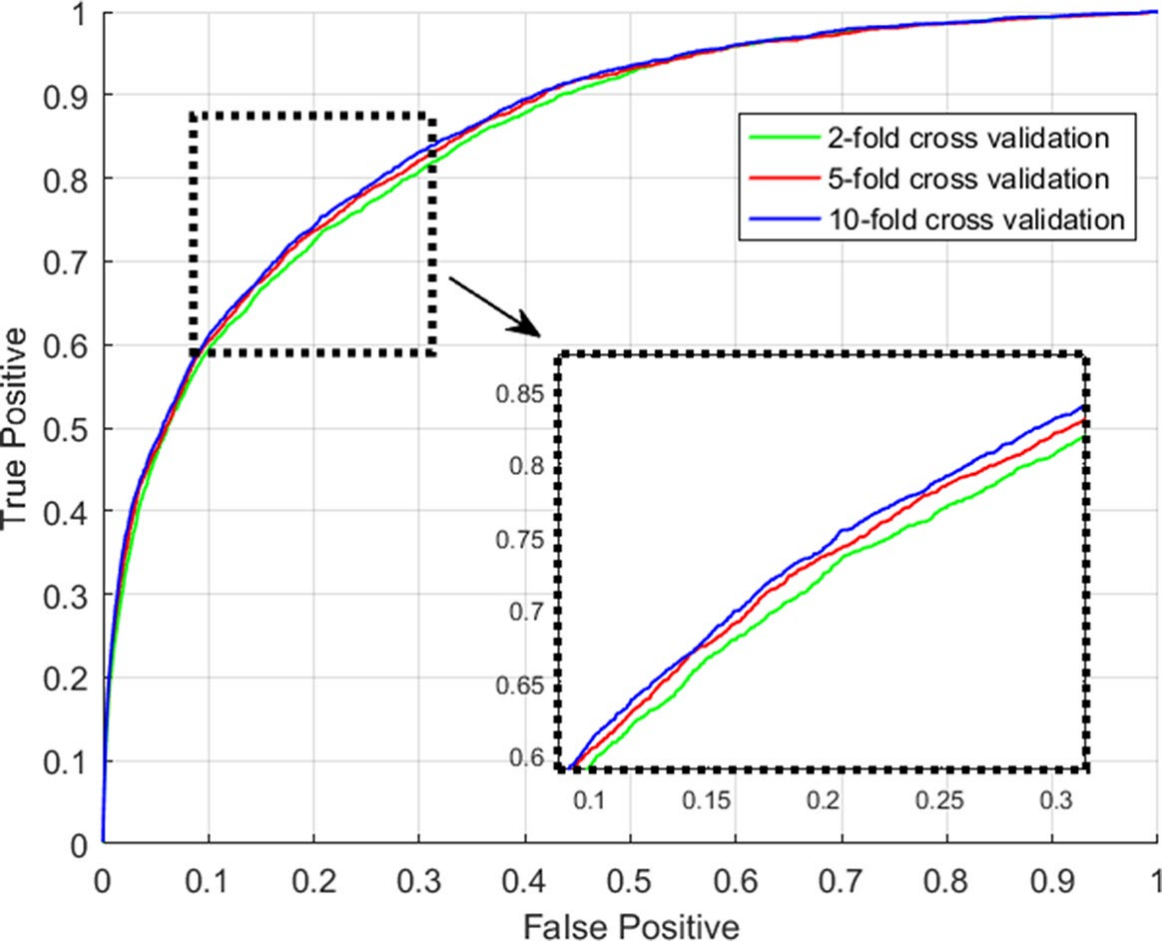
The ROC curves yielded by GCLMI on 2-fold, 5-fold and 10-fold cross validation.

**Figure 4:**
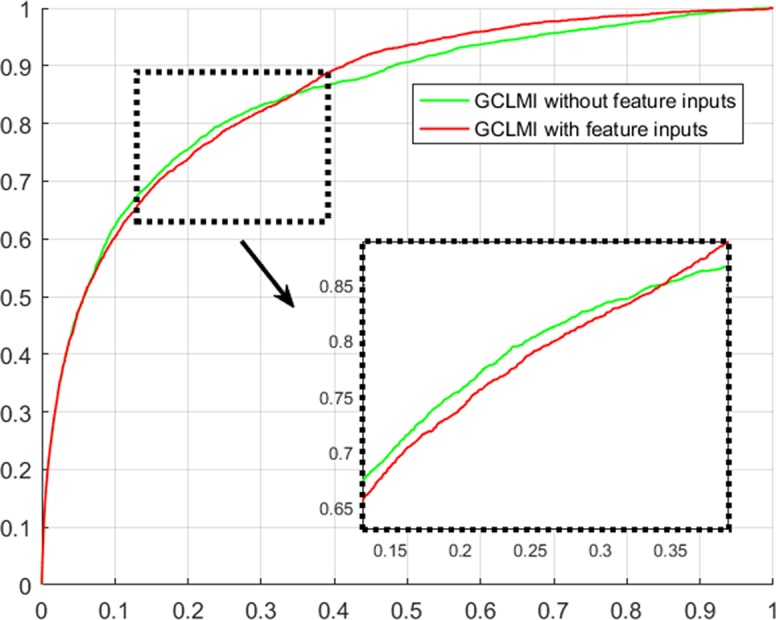
Evaluation of graph convolution layer w.r.t ROC curves on 5-fold cross validation.

Using techniques of graph convolution, spectral filter function integrates the information of attribute feature of input node with that of its neighbor nodes on the graph. GCLMI model uses graph convolution to build a data pre-processing module so that it can train the embedding features of nodes in an end-to-end learning manner. In this section, we evaluate the effectiveness of graph convolution with regard to its ability to integrate the raw data of input feature. Specifically, we compare the standard pipeline of GCLMI with the case that the input features are removed. To this aim, each entry of the input feature matrix A in formula 7 is replaced with the value of 1. In this case, the operator of graph convolution would be meaningless as all node features are the same. We implemented such modified computation process in 5-fold cross validation. As a result, without any input of node feature, the GCLMI model yield an AUC of 0.8483 on the 5-fold cross volition experiment, significantly lower than AUC of 0.8567 yield by the standard computational pipeline (see **Figure 3**). The result shows that the graph convolution designed in the model of GCLMI is feasible and effectively to integrate the raw data of feature inputs (see **Figure 4**).

### Evaluation of the Impact of Negative Sampling

There is still no biological experiment confirming any lncRNA-miRNA pair that are definitely not interactive so that no database can provide the data of negative samples for our training. For this reason, the prediction task in this work can be considered as a semi-supervised learning problem. Considering the known lncRNA-miRNA network is sparse, sampling on the unlabeled samples could generate a data source in which underlying negative samples are involved. Information of unlabeled data can be properly leveraged to push the limits of poor data resource for training (Zheng et al., 2014). To do so, we implement negative sampling on the unlabeled samples in each training epoch to construct negative sample set for training. However, the number of samples from negative sampling can have an effect on the prediction performance of the proposed model. A larger amount of negative sample can provide data resource for training and good performance could be achieved with more information for model to learn. However, it can also cause the problem of unbalanced training data. In this view, the choice of the size of negative sample set is important for an accurate prediction of GCLMI model. In each training epoch, the size of negative sample set is fixed as a ratio p of that of positive samples. In this section, we explore the prediction performance of GCLMI with different values of p (i.e. 0, 0.5, 1.0, 3.0, 5.0, 10.0).

**Figure 5:**
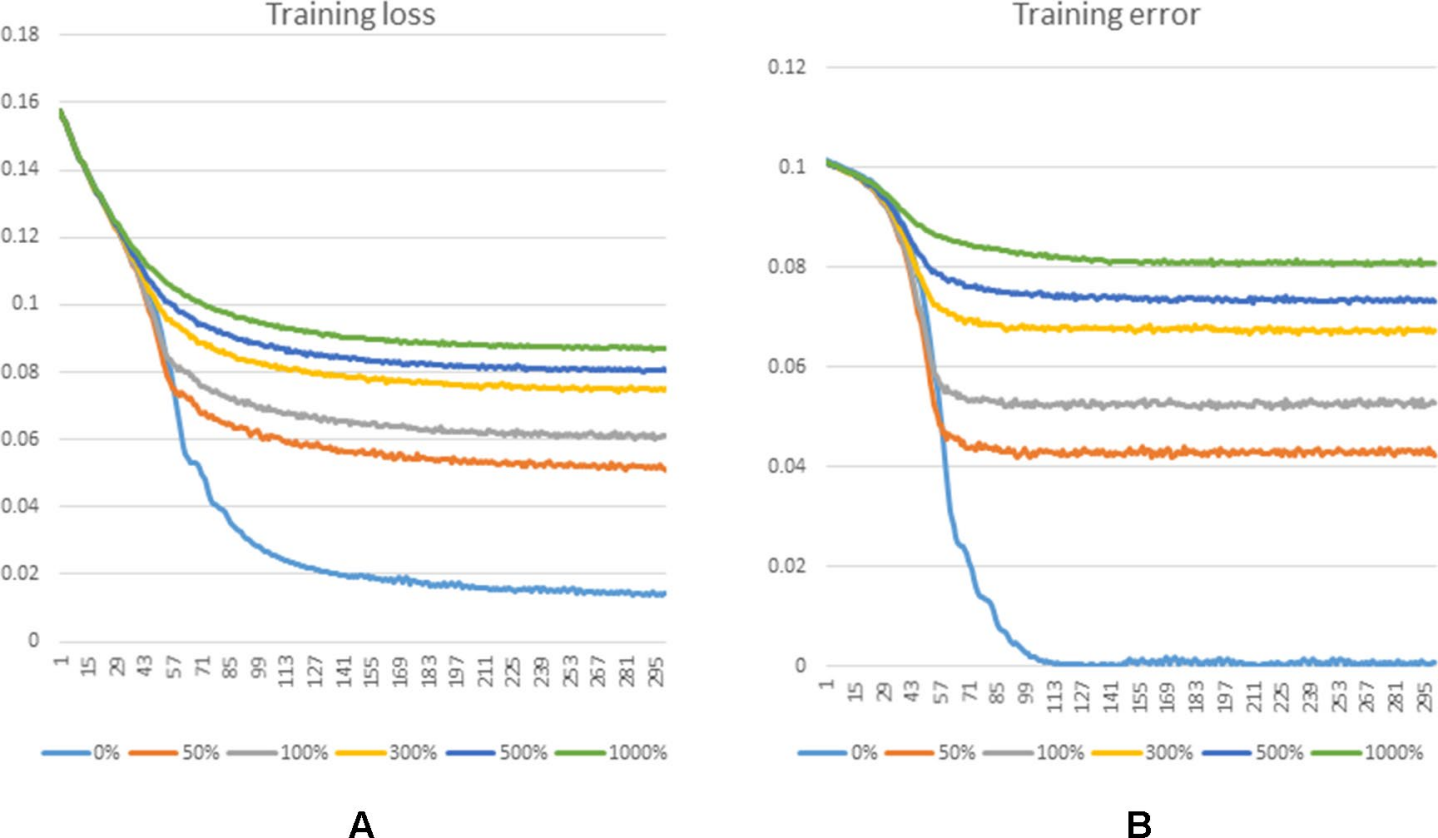
Training process of GCLMI in different training epochs with different negative sample sets. **(A)** and **(B)** illustrate the training loss and training error in training process, respectively.

**Figure 5** shows the training loss and training error along with increased training epoch in this series of experiments. We calculated the training loss and training error whose definitions are as the Equation 10 and the first term of Equation 10, respectively. The curves of **Figure 5(A)** and **Figure 5(B)** show that the training processes of GCLMI with different sizes of negative sample set are similar. For most of experiments, the corresponding training loss and training error could be convergent to their lower bounds before the 250th epoch and 150th epoch, respectively, illustrating the computational process is robust to different negative sampling. The prediction performance of GCLMI with different negative sampling is also evaluated. As shown in the **Figure 5**, the prediction performance varies with different sizes of negative sample set in term of the AUC value. Specifically, when the number of negative samples is set as 3 times of positive samples, the model achieves its highest prediction performance with AUC of 0.8567. It also should be noted that the prediction performance of GCLMI declines greatly with p set as 0. As setting p = 0 means that no negative sample is used for training, this result illustrates that negative sampling is effective and necessary for an accurate prediction of large-scale lncRNA-miRNA interactions.

### Prediction Performance of GCLMI on k-Fold Cross Validation

For the evaluation of the performance of our proposed model with regard to the prediction accuracy on lncRNA-miRNA interactions, we adopt the evaluation frameworks of 2-fold, 5-fold and 10-fold cross validation. All experiments in this work are conducted on a real dataset involving experimentally-confirmed lncRNA-miRNA interactions. Specifically, in the k-fold cross validation, all known lncRNA-miRNA interactions are roughly divided into k parts, each of which is used as testing sample set in turn and the rest is used as training sample set. After implement the prediction process of GCLMI with training set as input, each testing sample obtain its prediction score presenting the confidence coefficient about the link existence. We consider all the 209,152 unlabeled lncRNA-miRNA pairs as candidate samples and compute the ranks of prediction scores among the candidates.

**Figure 6:**
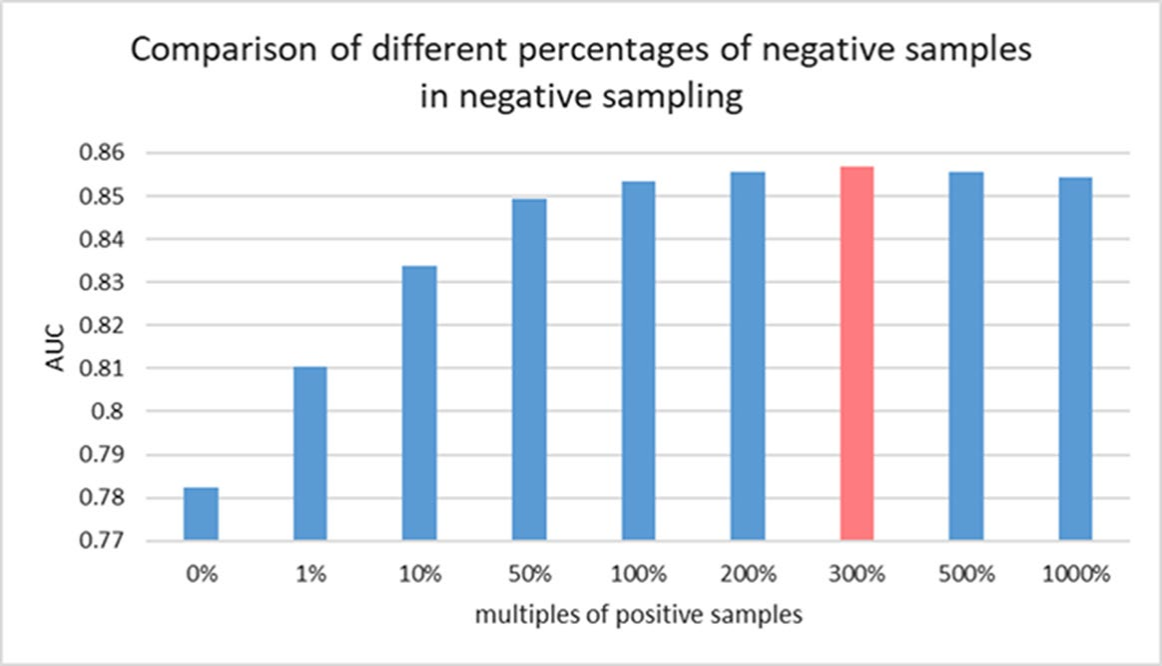
Comparison of prediction performance of GCLMI with different negative sample sets.

We consider those testing samples with a higher rank than a given threshold as positive. By setting different threshold in the experiments, we compute the corresponding true positive rates (TPRs, sensitivity) and FPRs (1-specificity) for each threshold. Specifically, given a threshold, sensitivity denotes the percentage of testing samples with higher ranks and specificity is the percentage of testing sample with lower ranks. Based on TPRs and FPRs, the corresponding ROC curve (receiver operating characteristic curve) is plotted and the area under the curve (AUC) is computed as a main evaluation criterion for the performance. The value of AUC lies between 0.5 and 1, where 0.5 means a purely random guess and 1 denotes a perfect prediction. As some of known lncRNA-miRNA interactions take turns to be used as testing samples and assumed to be unknown in the prediction process, if they obtained a high rank among those unlabeled samples in general, it means the prediction performance is good and prediction model is feasible. In addition, as the division of sample sets is random, we repeat the sampling implement GCLMI model with different sample division 20 times to avoid the bias caused by such partition. The standard deviation is also calculated for each cross validation. As a result, conducting GCLMI on the collected dataset, we obtain good prediction performance with average AUCs of 0.8492+/−0.0013, 0.8567+/−0.0009 and 0.8590+/−0.0005 in 2-fold, 5-fold and 10-fold cross validation, respectively (see **Figure 6**). As shown in **Table 1**, the increase of fold number in cross validation boosts the performance of GCLMI because more data resource in training set would benefit the prediction performance. In this view, we anticipate that GCLMI model is able to yield more reliable results with more ground true input data in the future. The results of high AUCs illustrate the reliable performance for predicting lncRNA-miRNA interaction on a large scale.

**Table 1:** Prediction performance w.r.t. AUC in 2-fold, 5-fold and 10-fold cross validation.

Cross validation	2-fold CV	5-fold CV	10-fold CV
Average AUC	0.8492 + /-0.0013	0.8567 + /-0.0009	0.8590+/-0.0005

### Performance Comparison With Other Similarity-Based Methods

Current approaches to predict new links on biological bipartite networks are mainly based on similarity-based assumption (Sun et al., 2018). Given a network in which two types of nodes representing two kinds of research objects are involved, most of previous prediction model assumes that similar objects of one type tend to be associated with those of another type (Huang et al., 2016b). Therefore, their prediction performance could be greatly influenced by the measurement they adopt to calculate the similarity scores among object of the same types (Huang et al., 2017b). For example, KATZHMDA model calculates the similarity of microbes using the Gaussian kernel and EPLMI model uses Person correlation coefficient for the similarity of lncRNA and miRNA based on their expression profiles (Huang et al., 2016a; Huang et al., 2018). However, such linear computation method may so simple that it fails to describe the general similarity of lncRNA or miRNA with regards to their roles in regulation network based on their expression profile. To bypass such barrier, we propose an end-to-end prediction model using graph convolution technique and therefore the prediction is free of any calculation for similarity.

To further evaluate the prediction performance, several similarity-based methods are implemented on the same dataset for performance evaluation, using the same similarity matrices of lncRNA and miRNA based on Person correlation coefficients of expression profiles. The comparison methods include two types of neighbor-based collaborative filtering (i.e. lncRNA-based CF and miRNA-based CF), matrix factorization-based method (i.e. SVD-based CF and basic latent factor model) and EPLMI. Using 5-fold cross validation on the same dataset, the comparison result shows that the proposed model has the best prediction ability among five comparison methods with highest AUC values of 0.8567+/−0.0009 (see **Table 2**). We consider such superior in performance on link prediction is benefited from the end-to-end learning approach as GCLMI model was designed. It is anticipated that such end-to-end prediction model as we proposed would yield more accurate prediction results with a larger amount of high-dimension data as inputs in the future.

**Table 2:** Performance comparison among different methods by using RNA expression profile-based similarity in the framework of 5-fold cross validation.

Method	5-fold cross validation
lncRNA-based CF	0.6359 + /−0.0024
miRNA-based CF	0.8235 + /−0.0015
SVD-based CF	0.4967 + /−0.0340
Katz-based method	0.7439 + /−0.0017
Basic latent factor model	0.8253 + /−0.0024
EPLMI (Huang et al., 2018)	0.8447 + /−0.0017
The proposed model	0.8567 + /−0.0009

## Conclusion

Increasing evidence show that lncRNA and miRNA collaborate to form a regulation network for gene regulation. Interactions between lncRNA and miRNA thus provide great insights into understanding the molecular mechanism of the initiation and development of various types of complex diseases. However, little effort has been made to develop computational approach to predict lncRNA-miRNA interaction on a large scale. The main challenge comes from the small number of known interactions between lncRNA and miRNA (i.e. the sparsity of lncRNA-miRNA interaction network) and the limited understanding on the underlying pattern on lncRNA-miRNA interaction.

To address this issue, we proposed a deep learning-based prediction model named GCLMI which can effectively predict large-scale lncRNA-miRNA interactions. Given raw data as RNA attribute features, the GCLMI model is able to extract meaningful embedding features for both miRNA and lncRNA in an end-to-end training manner. The results of a series of experiments show that the low-dimension embedding learned from the proposed model is of good representation ability with regards to their relation on the interaction network. Benefited from the deep learning structure as GCLMI is designed, we anticipate that the proposed model could be used as a useful tool for an accurate prediction of large-scale lncRNA-miRNA interactions in the scenario that additional information describing features of lncRNA and miRNA is offered by the users. In the current version of GCLMI, other types of data relevant to intrinsic features of lncRNA and miRNA, such like ncRNA sequence information and structural data are still inapplicable for GCLMI to handle with, as the graph convolution operator needs numerical data as inputs. In the future, we will investigate solutions about this limitation.

## Author Contributions

Y-AH and Z-AH conceived the algorithm, developed the program, and wrote the manuscript. Z-HY and ZZ helped with manuscript editing, designed and performed experiments. Y-AH and Z-AH prepared the data sets, carried out analyses and helped with program design. All authors read and approved the final manuscript.

## Funding

Y-AH was supported by the National Natural Science Foundation

of China under Grant No. 61702424 and the Natural Science Basic Research Plan in Shaanxi Province under Grant No. 2018JQ6015. Z-HY was supported by the National Natural Science Foundation of China under Grant No. 61572506.

## Conflict of Interest Statement

The authors declare that the research was conducted in the absence of any commercial or financial relationships that could be construed as a potential conflict of interest.
